# TRIM44 promotes proliferation and metastasis in non-small cell lung cancer via mTOR signaling pathway

**DOI:** 10.18632/oncotarget.8586

**Published:** 2016-04-05

**Authors:** Ying Xing, Qingwei Meng, Xuesong Chen, Yanbin Zhao, Wei Liu, Jing Hu, Feng Xue, Xiaoyuan Wang, Li Cai

**Affiliations:** ^1^ The Fourth Department of Medical Oncology, Harbin Medical University Cancer Hospital, Harbin, China

**Keywords:** TRIM44, non-small cell lung cancer, metastasis, epithelial-mesenchymal transition, mTOR signaling pathway

## Abstract

Tripartite motif-containing protein 44 (TRIM44) was recently identified as a potential therapeutic target in several types of malignancy, but its effect on the clinical course of malignancy and its underlying regulatory mechanism remain largely unknown. The present study shows that upregulation of TRIM44 is associated with poor differentiation, advanced pTNM stage, adenocarcinoma subtype, lymph node metastasis and, most importantly, unfavorable survival in patients with non-small cell lung cancer (NSCLC). TRIM44 knockdown inhibited the invasion and migration of human NSCLC cells, which was concurrent with downregulation of mesenchymal markers and upregulation of epithelial markers. Overexpression of TRIM44 induced the epithelial-to-mesenchymal transition (EMT) and increased the metastatic potential of lung cancer cells. Additionally, TRIM44 induced cell proliferation *in vitro* and tumor growth *in vivo* by accelerating G1/S transition via upregulation of cyclins and CDKs. TRIM44-induced mTOR signaling, EMT, and cyclin/CDK upregulation were reversed by treatment with a mammalian target of rapamycin (mTOR) inhibitor. These results provide a model for the relationship between TRIM44 expression and lung cancer progression, and open up new avenues for the prognosis and therapy of lung cancer.

## INTRODUCTION

Lung cancer is the leading cause of cancer-related death worldwide, with nearly 1.4 million deaths recorded annually [1]. Non-small cell lung cancer (NSCLC) accounts for 70–80% of all lung cancer cases, and nearly 50% of patients with stage I NSCLC die within 10 years after initial diagnosis [2]. Even in early stage patients treated by radical surgical resection, the risk of recurrence is high [3]. Therefore, major efforts have been made to identify molecular markers that predict prognosis, and more new treatment strategies are needed [4, 5].

Recent studies indicate that some members of the tripartite motif (TRIM) protein family function as important regulators of carcinogenesis [6]. Of these, TRIM44 contains a B-box domain, a coiled-coil domain, and a zinc-finger domain that is also found in members of the ubiquitin-specific protease (USP) family [7]. TRIM44 may function as a “USP-like TRIM” to inhibit ubiquitination and subsequent biological events. To date, several studies have reported that TRIM44 contributes to diverse pathological conditions such as cancer, developmental disorders, neurodegenerative diseases, and viral infections [7–18].

The role of TRIM44 mRNA, protein, and activity in the development and progression of several malignant tumors has been examined [11–16]. Although TRIM44 facilitates the migration and invasion of human cancer cells [15, 16], the mechanisms underlying TRIM44 activity during lung cancer metastasis are unclear.

The results of the present study suggest that TRIM44 expression is predictive of NSCLC lymph node metastasis and poor survival. Also, we found that TRIM44 is required to maintain the aggressive and malignant phenotypes of lung cancer cells, and that TRIM44 increases EMT and cell cycle progression in tumor cells by activating the mTOR pathway. Together, the *in vitro* and *in vivo* experimental data allowed us to propose a new model for how TRIM44 promotes lung cancer progression.

## RESULTS

### TRIM44 expression in NSCLC tissues

**Figure 1:**
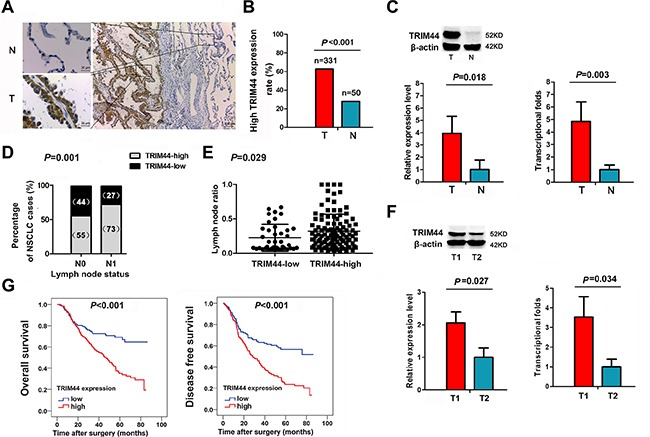
Increased TRIM44 expression in NSCLC patients is associated with lymph nodes metastasis and poor survival (**A**) Representative immunohistochemistry (IHC) images from a single NSCLC case (T) and matched adjacent normal lung tissue (N). The expression of TRIM44 protein in tumor tissues was significantly higher than that in adjacent normal lung tissues. (**B**) Histogram showing pooled data derived from NSCLC (T, *n* = 331) and normal lung (N, *n* = 50) tissues. *P* values were calculated using the χ^2^ test. (**C**) Representative western blot showing TRIM44 expression in lung tissues and a histogram showing pooled data from NSCLC (T, *n* = 20) tissues and adjacent normal lung tissues (N, *n* = 20) (left panel). Histogram showing TRIM44 mRNA expression in NSCLC (T, *n* = 20) tissues and adjacent normal lung tissue (N, *n* = 20) (right panel). Data are expressed as the mean ± SEM (*n* = 3). *P* values were calculated using Student's *t*-test. Normalization: The TRIM44/actin ratio was first calculated and normalized to 1.00. (**D**) Patients were classified in two groups, i.e., with (N1) or without (N0) lymph node metastasis. IHC analysis showed that 55% of patients without lymph node metastasis had high TRIM44 expression, whereas 73% of patients with lymph node metastasis had high TRIM44 expression. *P* values were calculated using the χ^2^ test. (**E**) Analysis of the lymph node ratio (the ratio of the number of metastatic lymph nodes to the total number of examined lymph nodes) in NSCLC. *P* values were calculated using Student's *t*-test. (**F**) Representative western blot showing TRIM44 expression in primary tumor tissues with lymph node metastasis and in those without lymph node metastasis (left panel). The levels of TRIM44 mRNA were quantified by real-time qRT-PCR (right panel). The histogram shows pooled data obtained from primary tumor lesions with lymph node metastasis (T1, *n* = 10) or without lymph node metastasis (T2, *n* = 10). Data are expressed as the mean ± SEM (*n* = 3). *P* values were calculated using Student's *t*-test. Normalization: The TRIM44/actin ratio was first calculated and normalized to 1.00. (**G**) High TRIM44 levels are associated with shorter survival in patients with NSCLC. Kaplan–Meier curves showing OS and DFS for patients with high and low TRIM44 expression.

IHC analysis revealed that TRIM44 was clearly localized to the cytoplasmic compartment of tumor cells (Figure 1A, Supplementary Figure S1). TRIM44 was highly expressed in 62.8% of NSCLC cases (208/331). High expression of TRIM44 was less frequent in squamous cell carcinoma (SCC) cases than in adenocarcinoma (ADC) cases (52.3% *vs.* 72.2%, respectively; *P* < 0.001; Table 1).

**Table 1:** Association between TRIM44 expression and clinicopathological characteristics of NSCLC patients

Variable	All patients (*n* = 331)	TRIM44 expression		*P*
		High (%) (*n* = 208)	Low (%) (*n* = 123)	
Smoking				
Never	125	76 (60.8)	49 (39.2)	0.550
Ever	206	132 (64.1)	74 (35.9)	
Gender				
Male	236	147 (62.3)	89 (37.7)	0.743
Female	95	61 (64.2)	34 (35.8)	
Age (years)				
< 60	189	122 (64.6)	67 (35.4)	0.458
≥ 60	142	86 (60.6)	56 (39.4)	
Differentiation				
Well	58	30 (51.7)	28 (48.3)	0.023*
Moderate	153	92 (60.1)	61 (39.9)	
Poor	120	86 (71.7)	34 (28.3)	
Histological cell type				
Adenocarcinoma	176	127 (72.2)	49 (27.8)	< 0.001*
Squamous cell carcinoma	155	81 (52.3)	74 (47.7)	
pStage				
I	134	70 (52.2)	64 (47.8)	0.004*
II	93	64 (68.8)	29 (31.2)	
III/IV	104	74 (71.2)	30 (28.8)	
pT classification				
T1	86	52 (60.5)	34 (39.5)	0.413
T2	218	136 (62.4)	82 (37.6)	
T3/4	27	20 (74.1)	7 (25.9)	
Lymph node metastasis				
Present	144	105 (72.9)	39 (27.1)	0.001*
Absent	187	103 (55.1)	84 (44.9)	
Adjuvant therapy				
Yes	188	119 (63.3)	69 (36.7)	0.843
No	143	89 (62.2)	54 (37.8)	
pT classification (patients with tumor invasion front no. = 50)				
T1	13	4	9	0.035*†
T2	32	14	18	
T3/4	5	4	1	

Abbreviations: NSCLC = non-small cell lung cancer; pTNM stage = tumor, node, metastasis (pathological stage); pT = pathological T stage; *n* = number of patients. *P < 0.05 was considered statistically significant. † Fisher's exact test.

Expression of TRIM44 protein was significantly higher in tumor tissues than in adjacent normal lung tissues (Figure 1A). In addition, TRIM44 expression in NSCLC tissues was significantly higher than that in normal lung tissues (62.8% *vs*. 28.0%, respectively; *P* < 0.001; Figure 1B).

We next examined TRIM44 protein expression in fresh tumor and normal tissues by western blot analysis. TRIM44 was detected as a band of ~52 kDa. The western blotting results showed that the expression of TRIM44 protein was higher in NSCLC tissues (*n* = 20) than in normal lung tissues (*n* = 20) (*P* = 0.018; Figure 1C).

Expression of TRIM44 mRNA was then examined in tumor and normal tissues using real-time quantitative RT-PCR. The results showed that the mean relative expression of TRIM44 mRNA in tumor tissues was significantly higher than that in normal lung tissues; indeed, tumor tissues expressed ~4.8-fold more TRIM44 mRNA than normal tissues (*P* = 0.003; Figure 1C).

### Association between TRIM44 expression and lymph node metastasis in NSCLC samples

We next searched for an association between TRIM44 expression in NSCLC samples and known clinicopathological factors. IHC analysis confirmed that elevated TRIM44 expression was significantly associated with poor differentiation (*P* = 0.023), advanced pTNM stage (*P* = 0.004), ADC subtype (*P* < 0.001), and the presence of positive lymph nodes (*P* = 0.001; Table 1; Figure 1D). TRIM44 expression was not associated with pT classification in the total cohort, but its expression at the tumor invasion front was significantly associated with pT classification in 50 samples with an assessable front (Table 1).

Recent studies have shown that the lymph node ratio (LNR) *per se* is an independent prognostic factor for recurrence after resection of NSCLC [19]. Therefore, we also examined the LNR, which is the ratio of the number of metastatic lymph nodes to the total number of examined lymph nodes. We found that patients with high TRIM44 expression had a significantly higher LNR than patients with low TRIM44 expression (*P* = 0.029; Figure 1E).

To explore the role of TRIM44 in NSCLC invasion, we next examined its expression in 20 patients grouped according to lymph node metastatic status. The results showed that TRIM44 protein expression was higher in NSCLC tissues from patients with lymph node metastasis (*n* = 10) than in those from patients without lymph node metastasis (*n* = 10) (*P* = 0.027; Figure 1F). Consistent with this, the results revealed that the mean relative expression of TRIM44 mRNA in tumor tissues from patients with lymph node metastasis was higher than that in tumor tissues from patients without lymph node metastasis (*P* = 0.034; Figure 1F).

Additionally, we examined lymphatic metastasis foci and matched primary tumor lesions from 30 NSCLC patients showing high expression of TRIM44. Notably, TRIM44 cytoplasmic staining was strong in both lymphatic metastasis foci and primary foci, and was independent of ADC or SCC status (Supplementary Figure S2).

### TRIM44 protein expression predicts survival in NSCLC patients

To determine whether TRIM44 expression is an independent prognostic factor for overall survival (OS) and/or disease-free survival (DFS) in NSCLC, we performed univariate and multivariate Cox regression analyses (Supplementary Table S1). The results of univariate analysis revealed that poor differentiation, ADC subtype, advanced pTNM stage, the presence of positive lymph nodes, and TRIM44 overexpression were significant indicators of poor OS. Advanced pTNM stage (HR, 2.814; 95% CI, 1.460–5.423; *P* = 0.002) and TRIM44 overexpression (HR, 1.695; 95% CI, 1.149–2.501; *P* = 0.008) were independent predictors of OS. These results were confirmed by multivariate analysis. Univariate analysis also revealed that ADC subtype, advanced pTNM stage, the presence of positive lymph nodes, and TRIM44 overexpression were significant predictors of poor DFS. Multivariate analysis also showed that advanced pTNM stage (HR, 2.236; 95% CI, 1.260–3.968; *P* = 0.006) and TRIM44 overexpression (HR, 1.737; 95% CI, 1.240–2.433; *P* = 0.001) were independent predictors for DFS. Kaplan-Meier analysis demonstrated that high expression of TRIM44 predicts a poorer prognosis in terms of both OS (χ^2^ = 20.37; *P* < 0.001; Figure 1G) and DFS (χ^2^ = 22.29; *P* < 0.001; Figure 1G). A stage-stratified analysis demonstrated that high TRIM44 expression may be a prognostic indicator of Stage I and II NSCLC (Supplementary Figure S1B).

### TRIM44 knockdown suppresses cell motility, migration, and invasion

**Figure 2:**
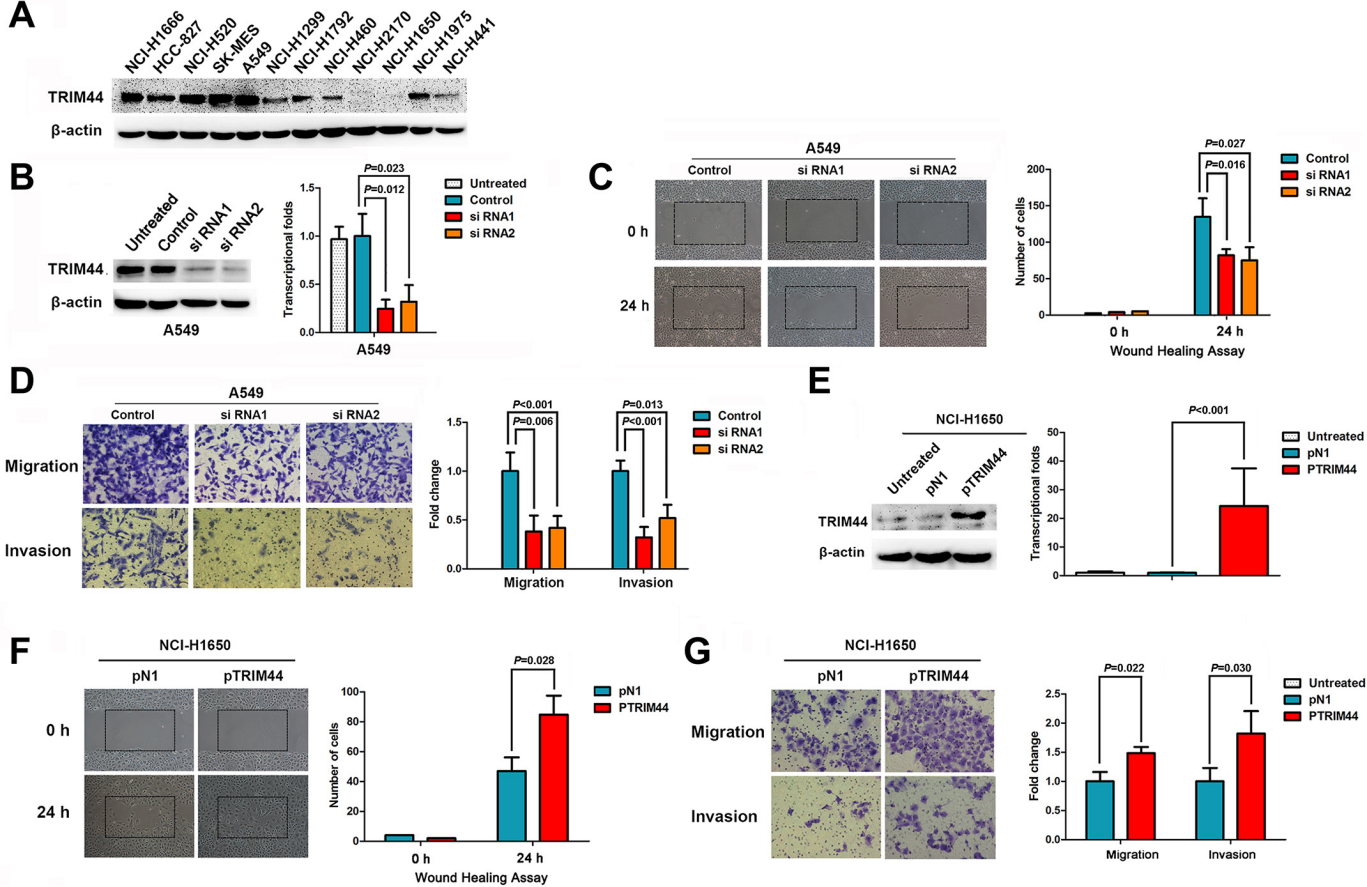
TRIM44 increases the motility and invasive properties of non-small cell lung cancer cells (**A**) Expression of TRIM44 in 12 lung cancer cell lines was examined by western blotting. β-actin was used as a loading control. (**B**) TRIM44 expression was confirmed by immunoblotting and real-time qRT-PCR. TRIM44 expression in A549 cells was reduced markedly by RNA interference. *P* values were calculated using Student's *t*-test (**C**) Wound healing assays were used to investigate the migration of A549 cells. *P* values were calculated using Student's *t*-test (**D**) Both invasion and migration of A549 cell lines (and their derivatives) were measured in a Transwell assay. *P* values were calculated using Student's *t*-test. (**E**) Immunoblot and real-time qRT-PCR analysis of TRIM44 protein and mRNA expression, respectively, in NCI-H1650 cells transfected with pDoubleEx-EGFP-TRIM44 (pTRIM44) or pDoubleEx-EGFP-N1 empty vector (pN1). *P* values were calculated using Student's *t*-test. (**F**) Wound healing assays were used to examine the migration of NCI-H1650 cells. *P* values were calculated using Student's *t*-test. (**G**) The migration and invasion of NCI-H1650 cell lines (and their derivatives) were measured in a Transwell assay. Data are expressed as the mean ± SEM (*n* = 3). *P* values were calculated using Student's *t*-test.

Western blot analysis detected TRIM44 protein in most of the lung cancer cell lines examined (10/12 cell lines; Figure 2A). Thus, A549 and NCI-H520 (ADC and SCC, respectively) cells were selected as a “loss-of-function” model because they expressed high levels of TRIM44. As shown in Figure 2, knockdown of TRIM44 protein and mRNA at 48 h after transient transfection of a TRIM44-specfic siRNA was more efficient than after transfection of control siRNA (Figure 2B; Supplementary Figure S3A).

Cell motility, migration, and invasion are necessary for cancer metastasis. Therefore, we next used a wound healing assay to test the effects of TRIM44 on NSCLC cell motility, migration, and invasion. The results showed that cells transfected with TRIM44-specific siRNA were slower to close scratch wounds than control cells (Figure 2C, Supplementary Figure S3B). In addition, a Transwell assay revealed that knocking down TRIM44 suppressed NSCLC cell migration and invasion when compared with control cells (Figure 2D, Supplementary Figure S3C).

### TRIM44 overexpression promotes cell motility, migration, and invasion

The human NSCLC cell line NCI-H1650 was chosen as a “gain-of-function” model to further validate the effect of TRIM44 on the migratory and invasive behavior of NSCLC.

TRIM44 expression was significantly upregulated following transfection of pDoubleEx-EGFP-TRIM44 (pTRIM44) into NCI-H1650 cells (*P* < 0.001; Figure 2E). The wound healing assay results showed that cells transfected with pTRIM44 closed scratch wounds more quickly than control cells (*P* = 0.029; Figure 2F). Increased TRIM44 expression resulted in increased migration and invasion of NCI-H1650 cells when compared with controls (Figure 2G).

### TRIM44 alters the expression of epithelial and mesenchymal markers

**Figure 3:**
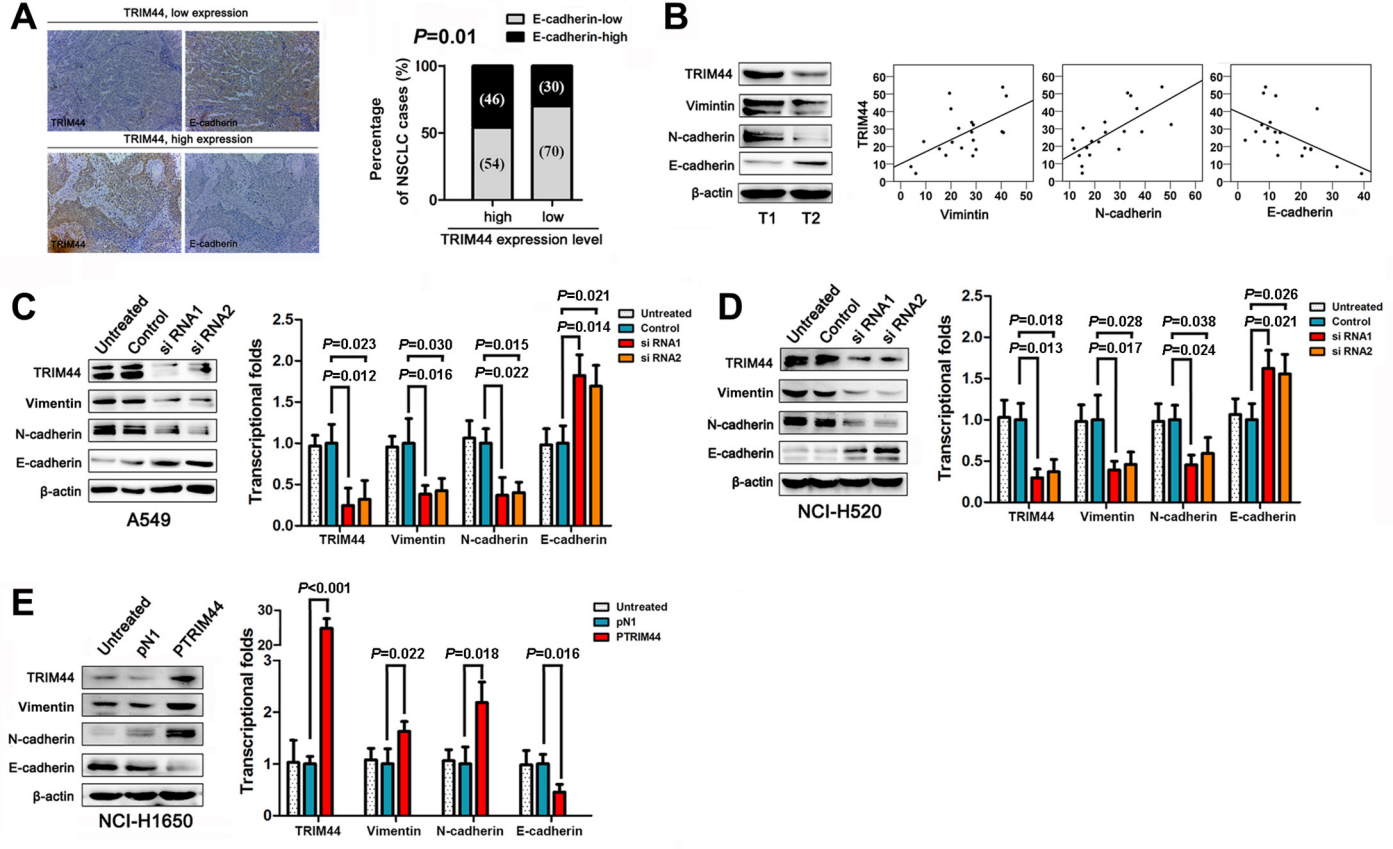
TRIM44 promotes NSCLC cell invasion and metastasis by increasing EMT (**A**) Representative images of TRIM44 and E-cadherin protein expression in the same tissues (left panel). E-cadherin expression patterns (high or low expression) were analyzed in NSCLC patients with high (*n* = 208) or low (*n* = 123) TRIM44 expression (two-sided Chi-square test) (right panel). (**B**) Representative western blot showing TRIM44, E-cadherin, N-cadherin, and Vimentin expression in NSCLC (T, *n* = 20) tissues (left panel). Scatter plot showing the correlation between TRIM44 expression and E-cadherin, N-cadherin, and Vimentin expression (right panel). **(C, D)** Western blot and real-time qRT-PCR analyses of E-cadherin, N-cadherin, and Vimentin expression in A549 and NCI-H520 cells treated with TRIM44-siRNA. *P* values were calculated using Student's *t*-test. (**E**) Western blot and real-time qRT-PCR analyses of E-cadherin, N-cadherin, and Vimentin expression in NCI-H1650 cells treated with pDoubleEx-EGFP-TRIM44 (p-TRIM44). *P* values were calculated using Student's *t*-test. All experiments were performed in triplicate, with three technical replicates.

Because loss of E-cadherin expression, a hallmark of EMT, is noted in many malignancies and is associated with increased metastatic potential [20], we examined E-cadherin levels by IHC analysis and assessed the relationship between E-cadherin and TRIM44 expression. High TRIM44 expression was significantly associated with loss of E-cadherin (*P* = 0.010; Figure 3A).

We next performed western blot analysis of 20 fresh tissue samples to examine the expression of TRIM44, N-cadherin, Vimentin, and E-cadherin. TRIM44 expression positively correlated with N-cadherin and Vimentin expression, but inversely correlated with E-cadherin expression (Figure 3B). Therefore, we measured the levels of E-cadherin, N-cadherin, and Vimentin at the protein level under conditions of aberrant TRIM44 expression. Knockdown of TRIM44 inhibited the expression of mesenchymal markers such as Vimentin and N-cadherin, but partially rescued the expression of epithelial markers such as E-cadherin (Figure 3C–3D). Conversely, overexpression of TRIM44 repressed E-cadherin expression and increased Vimentin and N-cadherin expression (Figure 3E). Similar correlations between TRIM44 and EMT markers were observed at the transcriptional level (Figure 3C–3E).

### TRIM44 increases the proliferation of NSCLC cells *in vitro* and tumor growth *in vivo*

**Figure 4:**
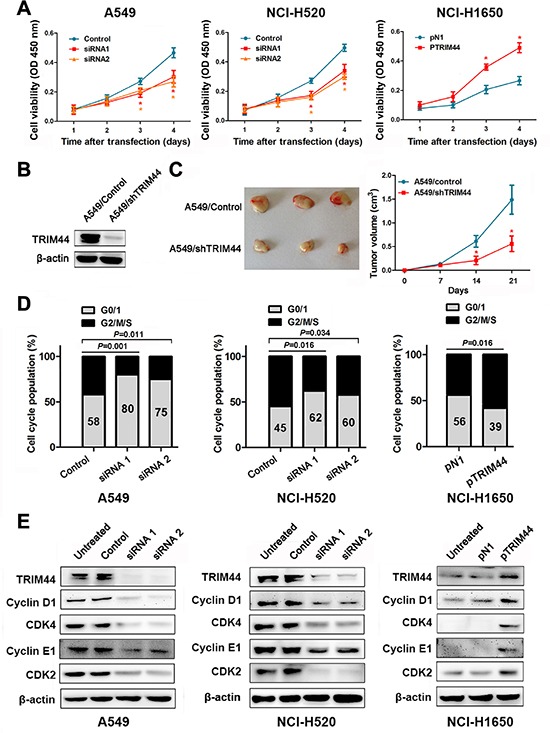
Effects of TRIM44 on NSCLC cell viability *in vitro* and *in vivo* (**A**) CCK-8 assays were used to examine changes in the proliferation rate of NSCLC cells at different time intervals (from 24 to 96 h). Data are expressed as the absorbance (mean ± SEM) for each group (*n* = 3). *P < 0.05 (Student's *t*-test). (**B**) Western blot analysis of TRIM44 and β-actin expression (loading control) in A549 cells stably transfected with shControl and shTRIM44 lentiviruses. (**C**) Knockdown of TRIM44 led to a marked reduction in tumor volume. The volume of tumors formed by stable A549/control or A549/shTRIM44 clones in NOD-SCID mice was measured weekly using a Vernier caliper. Left panel: gross view of isolated tumors. Right panel: shows mean ± SEM (*n* = 10) volume per group at each time point. *P < 0.05 (Student's *t*-test). (**D**) TRIM44 is involved in cell cycle progression. The cell cycle was examined by flow cytometry. *P* values were calculated using Student's *t*-test. (**E**) Representative western blot showing the effects of TRIM44 on the expression of cyclins and CDKs in NSCLC cells. All experiments were performed in triplicate, with three technical replicates.

The results of an *in vitro* proliferation assay showed that knockdown of TRIM44 significantly inhibited the proliferation of A549 and NCI-H520 cells when compared with control cells. As expected, the growth of NCI-H1650 cells transfected with pTRIM44 was greater than that of control cells (Figure 4A).

To investigate whether TRIM44 silencing reduces tumor growth *in vivo*, we established stable A549/control and A549/shTRIM44 clones with more than 80% efficiency (Figure 4B). Stable A549/control or A549/shTRIM44 clones were injected into the ventral region of NOD-SCID mice and tumor volumes were measured weekly. As shown in Figure 4C, knocking down TRIM44 in A549 cells led to marked growth suppression *in vivo*.

### TRIM44 accelerates the G1/S transition by upregulating cyclins and CDKs

Cell cycle control and cancer are intimately related because the cell cycle controls cell proliferation [21]. Therefore, we decided to analyze the effects of TRIM44 during specific phases of the cell cycle and examine its contribution to tumor cell proliferation. As seen in Figure 4D, siRNA-transfected cells arrested in G0/G1 phase, whereas pTRIM44-transfected cells showed accelerated entry into S phase. Next, we examined the expression of cyclins and CDKs after treatment with TRIM44 siRNAs or pTRIM44. We observed a positive correlation between TRIM44 expression and that of cyclins and CDKs, suggesting that TRIM44 plays an important role in regulating the G1/S transition (Figure 4E).

### Association between TRIM44 overexpression and mTOR activity

**Figure 5:**
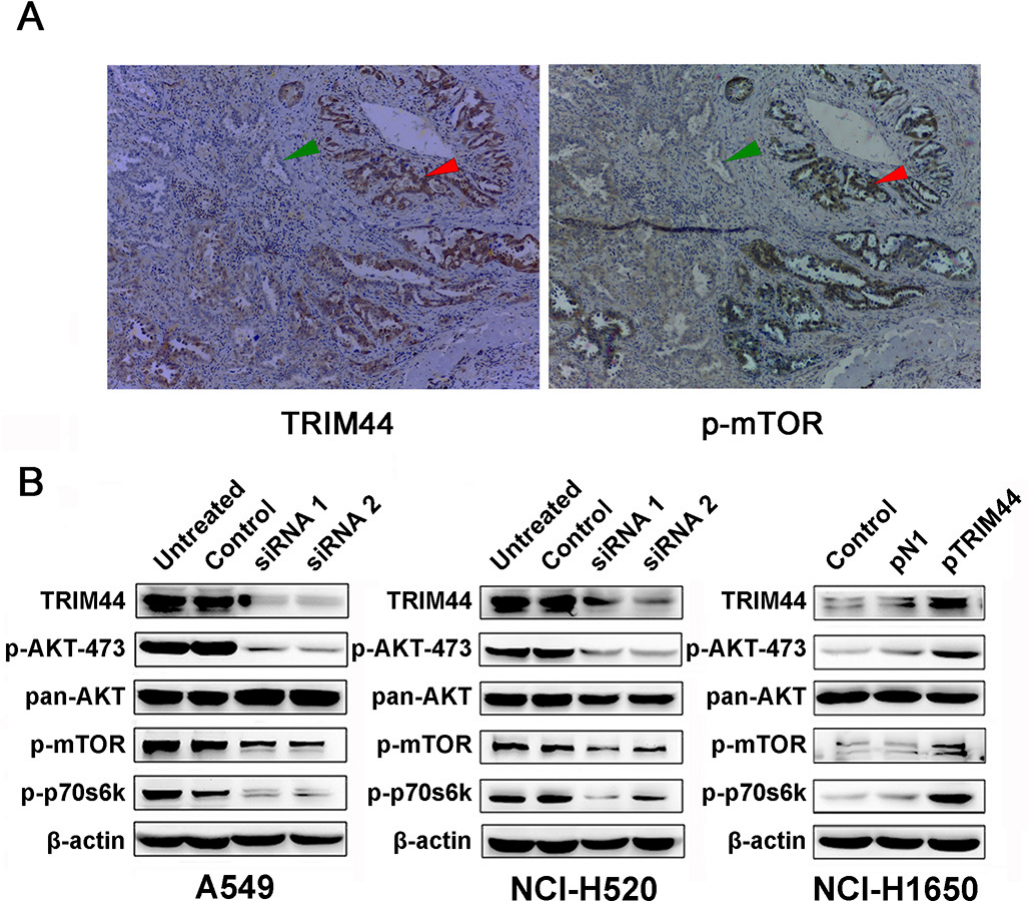
Association between TRIM44 overexpression and mTOR activity (**A**) Immunohistochemical analysis of TRIM44 and p-mTOR expression in primary tumor tissues from patient 1181. Red arrows show cells with high expression of TRIM44 and high mTOR activity, whereas the green arrows show cells with low TRIM44 expression and low mTOR activity. (**B**) Western blot analysis of TRIM44, mTOR signaling pathway components and downstream effectors in NSCLC cells.

The mTOR pathway is important for the regulation of cell proliferation, cell growth, and actin organization. A previous study reported an association between TRIM44 expression and mTOR signaling in esophagogastric cancers [13]; therefore, we reasoned that TRIM44 might regulate the mTOR pathway in NSCLC. Areas of tissue sections that staining strongly for TRIM44 also displayed high levels of mTOR activity (confirmed by staining for p-mTOR protein expression), whereas cells expressing low levels of TRIM44 also showed low levels of mTOR expression (Figure 5A). The phosphorylation of downstream mTOR substrates, including p-Akt (Ser473) and p-p70S6K (Thr389), in TRIM44-knockdown cells was markedly inhibited (Figure 5B). Consistent with this, the levels of p-AKT (Ser473) and p-p70S6K (Thr389) were also increased in TRIM44 overexpressing cells (Figure 5B).

### The TRIM44–mTOR axis increases metastasis and proliferation

Finally, to assess whether the metastasis and proliferation of NSCLC caused by TRIM44 resulted from activation of mTOR signaling, we transfected NCI-H1650 cells with pTRIM44 or the pDoubleEx-EGFP-N1 empty vector (pN1) and treated them (or not) with everolimus (an mTOR inhibitor).

**Figure 6:**
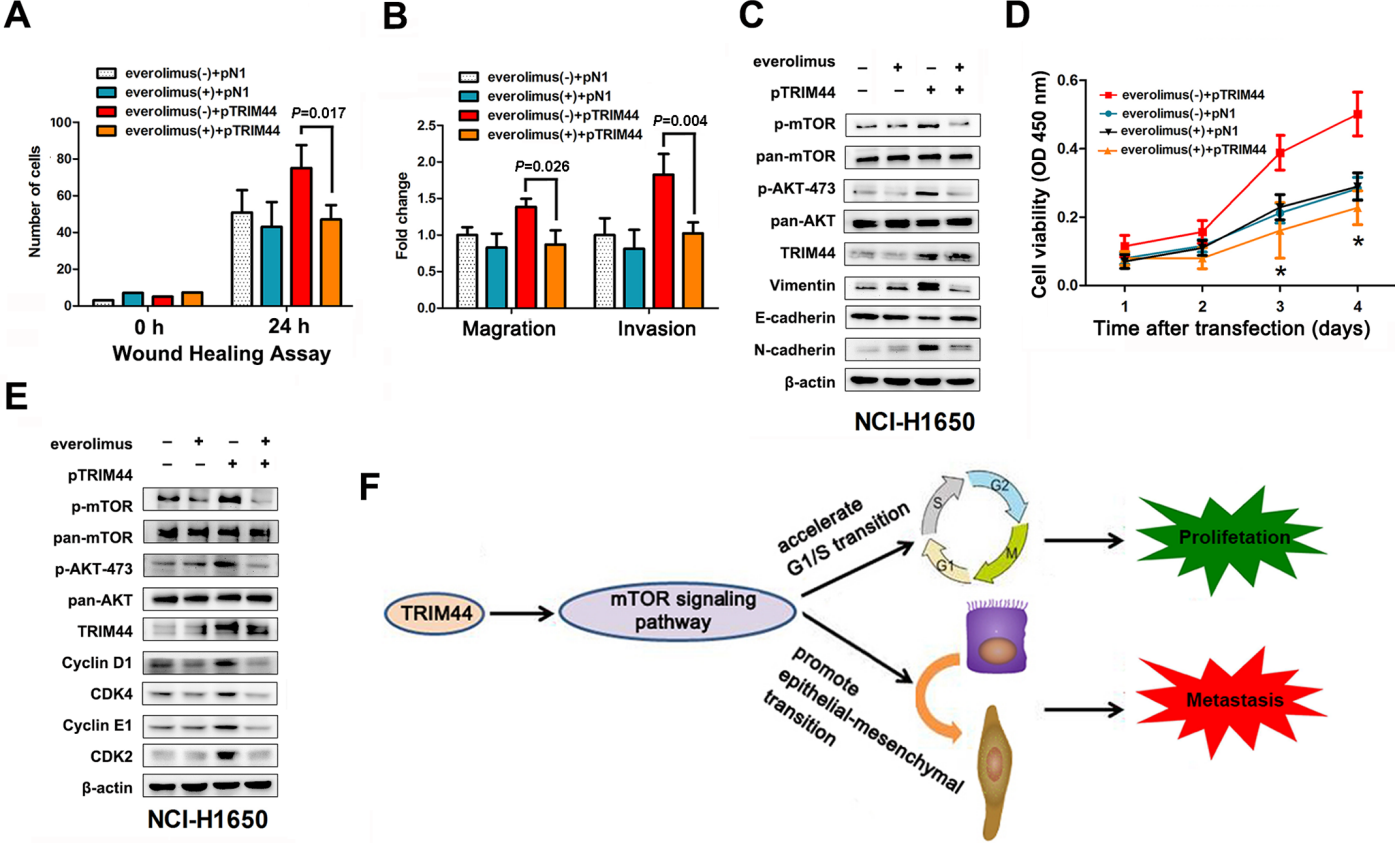
TRIM44 promotes metastasis and proliferation via the mTOR signaling pathway NCI-H1650 cells were transfected with p-N1 or p-TRIM44 prior to treatment (or not) with 2 mM everolimus as indicated. (**A**) Wound healing assays were performed to examine the migration of NSCLC cells. *P* values were calculated using Student's *t*-test. (**B**) Transwell assays were performed to investigate changes in the migratory and invasive ability of NSCLC cells. *P* values were calculated using Student's *t*-test. (**C**) Proliferation of NCI-H1650 cells was measured in a CCK-8 assay at different time intervals (from 24 to 96 h). All values represent the mean ± SEM of triplicate measurements. All experiments were repeated three times, with similar results. *P < 0.05 (Student's *t*-test). (**D**) Western blot analysis of components of the TRIM44/mTOR signaling pathway and EMT markers in NCI-H1650 cells. (**E**) Western blot analysis of components of the TRIM44/mTOR signaling pathway and cell cycle markers in NCI-H1650 cells. (**F**) Proposed model for TRIM44-induced malignant tumor progression. Increased expression of TRIM44 activates the mTOR signaling pathway, thereby accelerating the G1/S transition and promoting EMT with malignant changes leading to metastasis and proliferation.

As shown in Figure 6A and 6B, overexpression of TRIM44 induced NCI-H1650 cell motility, migration, and invasion; however, these behaviors were inhibited by everolimus. Similarly, western blot analysis of TRIM44 and EMT markers showed that EMT induced by upregulation of TRIM44 was also attenuated by everolimus (Figure 6C).

Also, everolimus reversed the increase in cell viability caused by TRIM44 (Figure 6D). Consistent with the role of the TRIM44-mTOR pathway in cell proliferation, everolimus also reduced the ability of TRIM44 to upregulate the expression of cyclins and CDKs (Figure 6E).

## DISCUSSION

Here, we report that the expression of TRIM44 was significantly increased in tumor tissues from patients with NSCLC. We also demonstrate for the first time that increased expression of TRIM44 is associated with poor differentiation, advanced pTNM stage, adenocarcinoma subtype, lymph node metastasis and, most importantly, poor survival. Thus, we speculate that TRIM44 is a key factor in promoting malignant progression of tumors. Overexpression of TRIM44 is frequently observed in esophagogastric cancer and is clinically relevant [13–15]. Consistent with our findings, other studies report that TRIM44 expression increases as Barrett's esophagus disease progresses to high-grade dysplasia and then to esophagogastric cancer [13, 14]. Elevated TRIM44 expression in gastric cancer correlates with lymph node invasion and recurrence and is a predictor of poor survival [15]. Similarly, breast cancer patients showing TRIM44 amplification have a significantly poorer prognosis than patients with normal copy numbers [13]. Collectively, these data suggest that TRIM44 is an important factor in the oncogenesis and progression of human malignancies. Although we feel that the results of the present study are intriguing, there are some limitations. For example, the current study was retrospective in nature and the number of study patients was small. Thus, a more thorough investigation in a larger series of patients is necessary to confirm the data.

In this study, we analyzed the association between TRIM44 expression and the pT classification in 50 samples with a tumor invasion front. A determination of the status of cells in the inner tumor mass may be inadequate for the pT classification of non-small cell lung tumors, because angiogenesis does not take place in the inner areas of these tumors, which slows the process of tumor growth [22, 23]. During tumor progression, tumor cells migrate into and invade the surrounding tissue, resulting in an invasive front, which may be more important for primary tumor growth than the inner parts of the tumor [23, 24]. Therefore, the TRIM44 staining level at the invasive front is a better measure of proliferative potential.

Metastatic disease is the primary cause of death in patients with NSCLC [25]. Tumor metastasis is a complex process, starting with primary tumor invasion through endothelial barriers via a process known as EMT, which is characterized by loss of cell-cell adhesion and increased cell motility [26, 27]. Preliminary functional studies show that siRNA knockdown of TRIM44 suppresses cancer cell migration and invasion [15, 16]; however, it is not known whether TRIM44 can induce tumor EMT. The present study is the first to demonstrate that TRIM44 represses the expression of E-cadherin and potentiates the EMT program in NSCLC cells.

A previous study showed that knocking down TRIM44 expression impaired the proliferation of human gastric cancer cells *in vitro*, but it did not fully elucidate the mechanism via which TRIM44 promotes cancer cell proliferation [15]. To the best of our knowledge, the present study is the first to directly demonstrate that high levels of TRIM44 promote NSCLC cell proliferation both *in vitro* and *in vivo*. Furthermore, we showed that TRIM44 accelerates the G1/S transition by upregulating cyclins and CDKs, thereby promoting cell cycle progression and cell proliferation. Thus, we have identified two novel functions for TRIM44, namely promotion of cell growth and cell cycle regulation.

In addition, we showed that TRIM44 overexpression leads to high mTOR activity, a finding supported by the reduced mTOR signaling in cancer cell lines after siRNA knockdown of TRIM44, and colocalization of TRIM44 with p-mTOR in patient samples [13]. We also showed that TRIM44 phosphorylates mTOR, suggesting that TRIM44 functions upstream of the mTOR signaling pathway. TRIM44 might deubiquitinate members of the TRIM family that contain a RING-finger domain function and function as E3 ubiquitin ligases, which ubiquitinated and degraded AMPK [7, 28]. AMPK inhibits mTOR signaling [28, 29], so TRIM44 might upregulate mTOR signaling. The molecular mechanism of TRIM44 regulation of mTOR signaling in NSCLC requires further study.

However, the role of the TRIM44-mTOR axis in the progression of human malignancies remains unclear. Emerging data show that mTOR increases both cell proliferation and motility in various species from yeast to human; thus, it is thought to be a key player in these biological processes [30–32]. Therefore, we also examined whether the TRIM44-mTOR axis increases EMT and cell cycle progression. As expected, an mTOR inhibitor reversed the effects of the TRIM44-mTOR axis, promoting EMT and upregulating the expression of cyclins and CDKs (Figure 6F). This suggests that increased mTOR signaling is required for NSCLC metastasis and proliferation caused by TRIM44. Taken together, the findings suggest that TRIM44 has a central role in the aggressive and invasive properties of tumors.

Here, we propose a model of the molecular mechanism underlying the effects of TRIM44 on lung cancer progression (Figure 6F). TRIM44 overexpression promotes the EMT program and, thus, tumor invasion/metastasis in NSCLC cells via the mTOR signaling pathway. Simultaneously, the TRIM44-mTOR pathway accelerates G1/S phase transition, thereby inducing cell proliferation.

Interestingly, other reports suggested that TRIM44 may function as a “USP-like TRIM” to regulate the deubiquitination and stabilization of oncogenes. This is because the N-terminal region of TRIM44 contains a zinc-finger domain, which is found in ubiquitin hydrolases (the ZF UBP domain). This domain is also present in USP20 and USP33 [33, 34]. Indeed, USP20 deubiquitinates and stabilizes its associated protein, hypoxia-inducible factor (HIF)-1α, resulting in the promotion of cancer cell proliferation, invasion, and metastasis [35, 36]. Therefore, the mechanism by which TRIM44 contributes to cancer progression is both important and interesting.

In light of the above findings, TRIM44 can be considered as a novel marker of lymph node metastasis in NSCLC patients. Furthermore, TRIM44 contributes to EMT and cell cycle progression by modulating the mTOR signaling pathway, thereby stimulating NSCLC cell metastasis and proliferation. This study not only reveals the pathological role of TRIM44 in NSCLC, but also suggests that it could be used as a prognostic factor and therapeutic target in NSCLC and, possibly, other cancers as well.

## MATERIALS AND METHODS

### Clinical specimen and cell lines

Clinical samples were obtained from 331 patients with NSCLC who were surgically treated at Harbin Medical University Cancer Hospital from January 2006 to December 2009. Thirty matched metastatic lymph nodes from 30 patients whose primary tumor expressed high levels of TRIM44 were selected for immunohistochemical (IHC) examination. Fresh tissues (paired NSCLC tumor samples and matched adjacent normal tissue samples) were resected from 20 NSCLC patients between June 2012 and June 2013. The 20 NSCLC tissues were assigned to one of two groups according to the lymph node metastasis status of the primary tumor (group T1: tumors with lymph node metastasis, *n* = 10; group T2: tumors without lymph node metastasis, *n* = 10). This study was approved by the Institute Research Medical Ethics Committee of Harbin Medical University. All of the patients gave their informed consent.

The human lung adenocarcinoma cell lines A549, NCI-H1666, NCI-H1975, NCI-H1299, NCI-H1792, NCI-H1666, NCI-H1650, PC9, and HCC827, the lung squamous cell carcinoma cell lines NCI-H520 and SK-MES, and the large cell carcinoma cell line NCI-H460 were purchased from American Type Culture Collection (ATCC, Manassas, VA), which employed short tandem repeat (STR) profiling to ensure cell line authenticity 3 months before the initiation of this study. No other forms of authentication were implemented by the author during the course of the study.

### Immunohistochemistry

Immunohistochemical analysis of TRIM44 was performed using the Two-Step IHC Detection Reagent (PV-6001) kit (Zhong Shan Golden Bridge Biological Technology Inc., Beijing, China), according to the manufacturer's instructions. Paraffin-embedded tissue blocks containing lung specimens were cut in a microtome (~4 μm thick) and stained with hematoxylin and eosin (H & E). In brief, tissue sections were deparaffinized in xylene and rehydrated in a series of graded alcohol solutions according to standard procedures. The sections were then immersed in 3% hydrogen peroxide for 10 min to remove endogenous peroxidase. Antigen retrieval was performed for 3 min in a pressure cooker containing 10 mM citrate buffer (pH 6.0) to enhance immunoreactivity.

The slides were then incubated with anti-TRIM44 (1:50; ProteinTech, Manchester, UK, 11511), anti-phospho-mTOR (Ser2448) (1:200; Cell Signaling Technology, Inc. USA; 5536) and anti-E-cadherin (1:50; Santa Cruz Biotechnology, CA, USA; sc-7870) at 4°C overnight. After washing with phosphate-buffered saline, a rabbit secondary antibody (Zhong Shan Golden Bridge Biological Technology Inc., Beijing, China) was applied and incubated for 20 min at room temperature. Color was developed using 3,3′-diaminobenzidine tetrahydrochloride (Dako, Hamburg, Germany). The slides were then counterstained with Meyer's hematoxylin and dehydrated in ethanol. Finally, the slides were mounted and cover-slipped with Resina. The negative control slides were stained with rabbit serum instead of primary antibodies. Human hepatocellular carcinoma sections positive for TRIM44 were used as the positive control. All the tissue sections were analyzed by two independent pathologists experienced in evaluating IHC, both of whom were blinded to the clinicopathological data. The staining results were scored according to the following criteria: (a) percentage of immunoreactive cells: 0 (0%), 1 (1–10%), 2 (11–50%), 3 (51–70%), or 4 (≥ 71%); and (b) staining intensity: 0 (negative staining), 1 (weak staining), 2 (moderate staining), or 3 (intense staining). The final score for TRIM44 expression was the sum of both scores; thus, the final score ranged from 0 to 7 [37]. For the purposes of statistical analysis, a final staining score of < 4 was defined as low expression, and a score ≥ 4 was defined as high expression. Any discrepancies between scores were reviewed by the two pathologists plus a senior pathologist until a consensus was reached.

### Cell invasion and migration assay

The invasion and migration assays were performed in 24-well FluoroBlok cell culture inserts (BD Biosciences) fitted with a PET membrane (8 μm pore size). The inserts were coated with 100 μL of Matrigel matrix (1 μg/μL; BD Biosciences) at 4°C overnight. Following starvation for 6 h in serum-free RPMI 1640 or DMEM, cells were harvested from a single sub-confluent 10 cm dish using cell dissociation buffer (Life Technologies), spun at 500 × g for 3 min, and resuspended in RPMI 1640. Cells (4 × 104 in 300 μL of RPMI 1640 or DMEM) were then seeded onto the insert and 700 μL of RPMI 1640 or DMEM supplemented with 10% FBS was added to the lower chamber of each Transwell. After incubating for 18 h at 37°C, the medium inside the insert was removed and the insert placed in a new 24-well plate. The cells present on the reverse side of the insert were then labeled with a fluorescent dye (Calcein AM; 4 μM in Dulbecco's PBS; BD Biosciences) for 1 h at 37°C.

### Cell proliferation assay

Cells were seeded in 96-well plates at a density of 7 × 10^3^ per well. After 24, 48, 72, or 96 h of growth, the cells were incubated with 10 μl of Cell Counting Kit-8 (CCK-8) assay reagent (Dojindo, Kumamato, Japan) for 1 h at 37°C and the absorbance was measured at 450 nm. The assay was conducted using five replicate wells per sample and three parallel experiments were performed.

### Xenograft models

Healthy purebred BALB/C nude mice were maintained according to the guidelines for the administration of laboratory animal research as outlined by the Institutional Animal Care and Use Committee of Harbin Medical University in China and the Care and Use of Laboratory Animals (National Institutes of Health, revised 1985).

To assess the effect of TRIM44 on tumor growth, A549/control or A549/shTRIM44 cells (5 × 10^6^ cells in 100 μL of PBS) were injected into the left (control) and right (shTRIM44) ventral regions of ten female NOD-SCID mice (6 weeks old). Tumor growth was monitored weekly by measuring the perpendicular tumor diameter (length (L) and width (W)) with a Vernier caliper. The tumor volume (V) was calculated using the formula: V = LW^2^/2. The research protocol was approved by the institutional ethics committee for the administration of laboratory animals of Harbin Medical University, China.

### Flow cytometry and cell cycle analysis

Cells were serum starved for 24 h to synchronize the populations at G0 before transfection. On Day 0, the serum-free medium was replaced with serum-containing medium for 48 h. Cells were then harvested and fixed overnight in 70% ethanol. Cells were then stained with FxCycle PI/RNase staining solution (KeyGEN BioTECH, Nanjing, China) and analyzed on an LSR II flow cytometer (BD Biosciences, San Diego, USA). Cell cycle analysis and model fitting was performed using FlowJo software (FlowJo LLC, Bethesda, USA).

### Statistical analysis

All analyses were performed using SPSS 19.0 for Windows (SPSS, Chicago, IL, USA). Student's *t*-test was performed for continuous variables and the χ^2^ test was used to analyze differences between categorical variables. Survival curves were plotted using the Kaplan–Meier method and compared using the log-rank test. Covariates that remained significant through univariate analysis were selected for multivariate analysis. The Cox proportional hazards model was used for the multivariate analysis of independent prognostic factors for OS and DFS. A two-sided *P* value < 0.05 was considered statistically significant.

## SUPPLEMENTARY MATERIALS FIGURES AND TABLES


