# Supplementary material for: TRIM44 promotes proliferation and metastasis in non-small cell lung cancer via mTOR signaling pathway
Source: Oncotarget. 2016 Apr 5;7(21):30479–91. doi: 10.18632/oncotarget.8586 (PMC5058694; doi:10.18632/oncotarget.8586)
Supplement: Supplementary file 1 [file oncotarget-07-30479-s001.pdf]

TRIM44 promotes proliferation and metastasis in non-small cell lung cancer via mTOR signaling pathway

Supplementary Materials

MATERIALS AND METHODS

Patients and cell cultures

The clinicopathological characteristics of the NSCLC patients are described in Table 1. The median age of the patients was 58 years (range, 28 to 82 years) and the mean age was 57.55 ± 8.914 years (mean ± s.d.). The histological type of lung cancer was defined according to the World Health Organization classifications, and primary cancers were evaluated in accordance with the American Joint Committee on Cancer (7th Ed.) staging system [1]. The study was approved by the Institute Research Medical Ethics Committee of Harbin Medical University. All patients provided informed consent. None of the patients received chemotherapy, radiotherapy, or immunotherapy prior to surgery.

Overall survival (OS) was defined as the time from surgery to the date of death. Disease-free survival (DFS) was defined as the time from surgery to the time of recurrence or death resulting from any cause. All NSCLC patients were followed up with until death or until the cut-off date (December 30, 2013). The mean and median follow-up time after discharge from hospital were 42.4 months and 46.8 months respectively, ranging within 2.5–86.5 months.

Cells were cultured according to the suppliers' instructions. Briefly, NCI-H1650 cells were grown in Dulbecco's modified Eagle's medium (DMEM; Invitrogen, Grand Island, NY) supplemented with 10% fetal bovine serum (FBS; Invitrogen). A549, NCI-H520, and NI-H460 cells were cultured in RPMI-1640 medium (Invitrogen) supplemented with 10% FBS (Hangzhou Sijiqing Biological Engineering Materials Co, Ltd, Hangzhou, China). NCI-H1975, NCI-H1299, NCI-H1792, NCI-H1666, NCI-H2170, HCC827, and PC9 cells were grown in RPMI-1640 medium (Invitrogen) supplemented with 10% FBS (Invitrogen).

RNA interference and plasmid transfection

Short interfering RNA (siRNAs) sequences specifically targeting human TRIM44 were synthesized by Invitrogen (Shanghai, China), as described previously [2] . The sequences were as follows: siRNA1, CACTTACGAAGCATACAACTA; siRNA2, TAGGATTGTCCTGACTCACTA; non-targeting control siRNA, UUCUCCGAACGUGUCACGUTT. Full-length human TRIM44 was cloned into the pDoubleEx-EGFP vector (Invitrogen) and the resulting construct designated pDoubleEx-EGFP-TRIM44 (pTRIM44). The pDoubleEx-EGFP-N1 empty vector (pN1) was used as a control.

For siRNA delivery, double-stranded RNA oligonucleotides were transfected using Lipo2000 (Invitrogen). A549 or NCI-H520 (grown to 30–50% confluence in six-well plates) were transfected with siRNAs (75 pmol per well) using 5 µL of Lipo2000 according to the manufacturer's instructions and then harvested 72 hours later for analysis.

Similarly, NCI-H1650 cells (30–50% confluent) were transfected with pTRIM44 or pN1 (2.5 µg per well) using 5 µL of Lipo2000 according to the manufacturer's instructions and then harvested 72 hours later for analysis.

Establishment of stable cell lines

To establish stable TRIM44 knockdown cell lines, the Lenti-shRNA vector system (pGCSIL-GFP) was constructed, packed, and purified by GeneChem (Shanghai, China) and manipulated according to the manufacturer's protocol. A TRIM44 short hairpin sequence (5′-TAGGATTGTCCTGACTCACTA-3′) was chosen. Briefly, shTRIM44 and shControl lentiviruses were used to infect A549 cells for 3 days. Stable clones were then selected with puromycin (1 µg/mL) and the knockdown efficiency determined by western blotting.

Wound healing assay

Cells were seeded in six-well plates and cultured in RPMI 1640 or DMEM containing 10% FBS until confluent. The cells were then scratched with a sterile 10 µL pipette tip to create artificial wounds. Phase-contrast images of the wound healing process were obtained digitally at 0 and 24 h after wounding using an inverted Olympus I × 50 microscope fitted with a 10× objective lens. Eight images per treatment were analyzed to determine average parameters with respect to the position of the migrating cells at the wound edges. Lines were digitally drawn using Image-Pro Plus software (Media Cybernetics, Rockville, USA).

Western blot analysis

Frozen tissue samples or lung cancer cells were homogenized in RIPA buffer containing a 1% protease inhibitor mixture. The mixture was centrifuged at 12 000 g for 15 min at 4°C and the supernatant obtained. Total protein was quantified using the Bradford method (Thermo Scientific, Waltham, MA, USA). Briefly, 30 µg of protein extract was separated by 12% SDS polyacrylamide gel electrophoresis (SDS-PAGE) and transferred onto PVDF membranes (Millipore Company, Billerica, MA, USA). The membranes were blocked with 2% bovine serum albumin (BSA) at 37°C for 1 h and incubated with the primary antibody overnight at 4°C. After washing, the membrane was incubated with a horseradish peroxidase-labeled secondary antibody for 1 h at room temperature and then washed again. The blots were stained using Super ECL Reagent kit (HaiGene, Harbin, China; M2301) and imaged using the FluorChem™ HD2 System (Proteinsimple, CA, USA). The antibodies used for western blotting are listed in Supplementary Table S2. The experiment was repeated three times.

RNA preparation and reverse transcription

Immediately following resection, fresh tissues were stored at –80°C until RNA extraction. Total RNA was extracted from fresh frozen samples or lung cells using Trizol reagent (Invitrogen, Carlsbad, CA), following confirmation by pathologists that the tumor samples contained at least 75% tumor cells. RNA quality and concentration were measured using a NanoDrop 2000 spectrophotometer (Thermo, Wilmington, DE, USA). Reverse transcription was performed using 2.0 µg total RNA in a 14.5-µL reaction mixture containing 2 × Power Taq PCR Master Mix kit (PR1702, BioTeke, Beijing, China), according to the manufacturer's instructions.

Real-time quantitative reverse transcriptase-PCR (qRT-PCR)

Real-time qRT-PCR was performed using the Exicycler96 Real-time RT-PCR system (A-2060, BIONEER, Daejeon, Korea) and SYBR Green Mastermix (SY1020, Solarbio, Beijing, China). The following TRIM44 primers were used: forward, 5′-GAGGAAGTGTGCCGAGAATG-3′; reverse, 5′-CTCTCCTGCTCCACCTTGAC-3′. The following β-actin primers were used as the internal reference: forward, 5′-CTTAGTTGCGTTACACCCTTTCTTG-3′; reverse, 5′-CTGTCACCTTCACCGTTCCAGTTT-3′. Amplification was performed under the following conditions: 95°C for 10 min, followed by 40 cycles of 95°C for 10 s, 60°C for 1 min, and 95°C for 15 s. Experiments were performed in triplicate. The results of the real-time qRT-PCR experiments were calculated using the $2^{-\Delta\Delta C(t)}$ method.

REFERENCES

1. Tsim S, O'Dowd CA, Milroy R, Davidson S. Staging of non-small cell lung cancer (NSCLC): a review. Respiratory Medicine. 2010; 104:1767–1774.
2. Ong CA, Shannon NB, Ross-Innes CS, O'Donovan M, Rueda OM, Hu DE, Kettunen MI, Walker CE, Noorani A, Hardwick RH, Caldas C, Brindle K, Fitzgerald RC. Amplification of TRIM44: Pairing a Prognostic Target With Potential Therapeutic Strategy. Journal of the National Cancer Institute. 2014; 106.

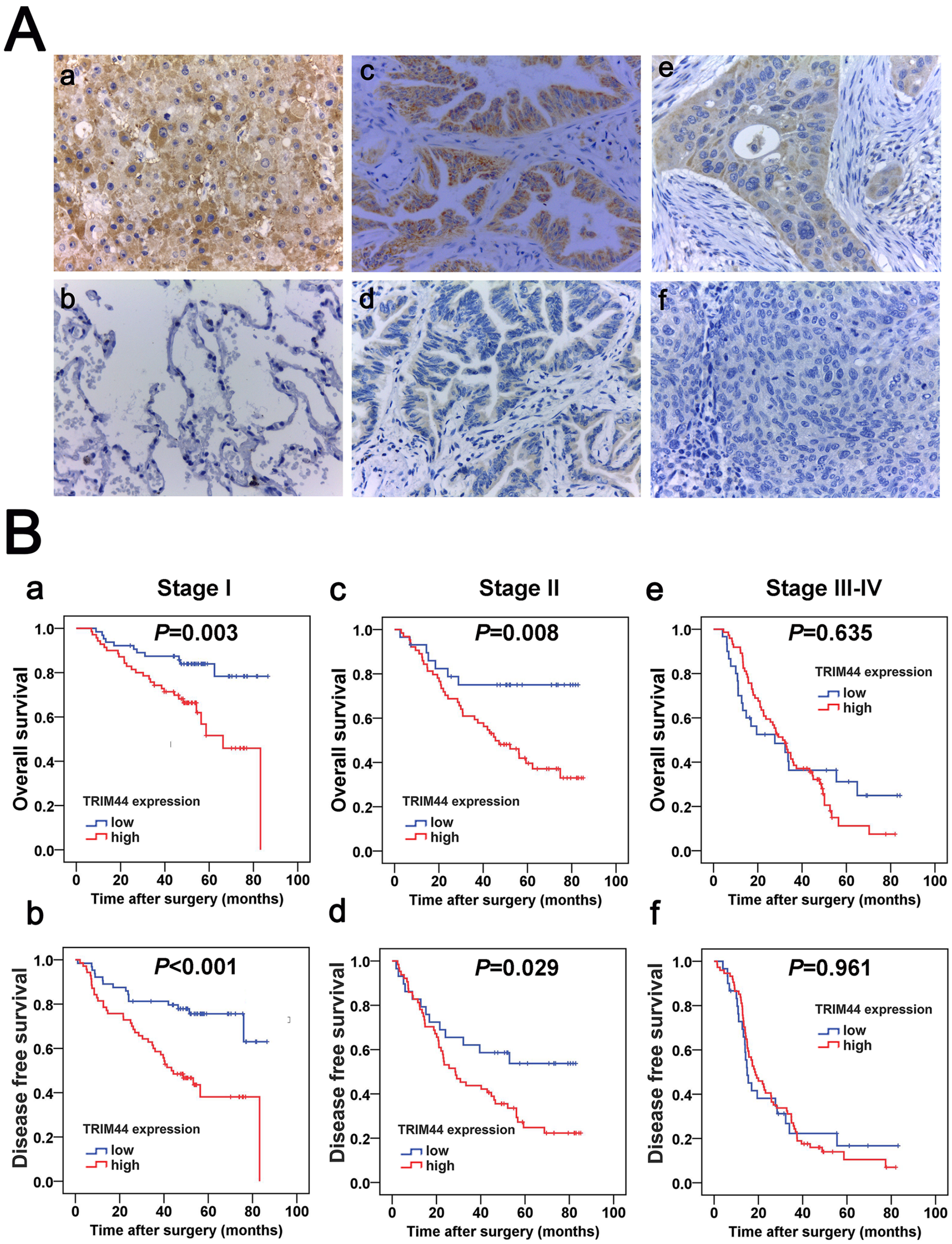

Supplementary Figure S1: (**A**) Representative photomicrographs showing immunohistochemical staining for TRIM44**.** (a) Positive control for hepatocellular carcinoma. (b) Negative staining of normal lung alveoli. (c) High expression in the ADC histotype. (d) Low expression in the ADC histotype. (e) High expression in the SCC histotype. (f) Low expression in the SCC histotype. (Original magnification, ×400). (**B**) Survival curves showing the correlation of TRIM44 with OS and DFS in NSCLC patients in different pStages. (a) Overall survival in stage I. (b) Disease-free survival in pStage I. (c) Overall survival in pStage II. (d) Disease-free survival in pStage II. (e) Overall survival in pStage III–IV. (f) Disease-free survival in pStage III–IV.

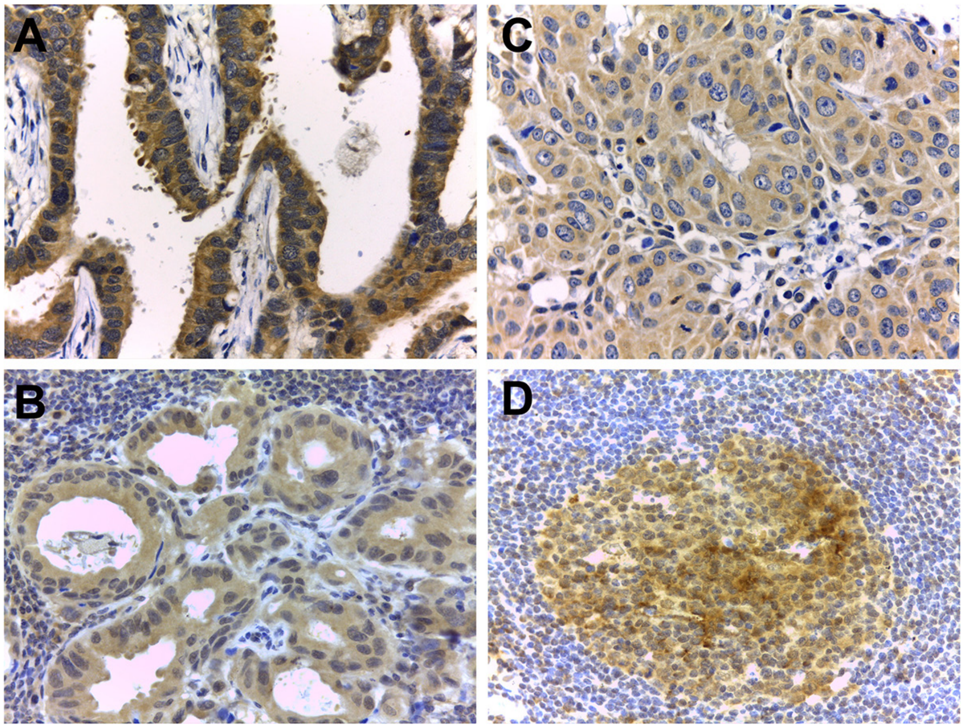

Supplementary Figure S2: TRIM44 expression in primary and metastatic lymph nodes from the same patient. (**A**) Primary lesion in the ADC histotype. (**B**) Metastatic lymph node in the ADC histotype. (**C**) Primary lesion in the SCC histotype. (**D**) Metastatic lymph node in the SCC histotype. (Original magnification, ×400). ADC: adenocarcinoma; SCC: squamous cell carcinoma.

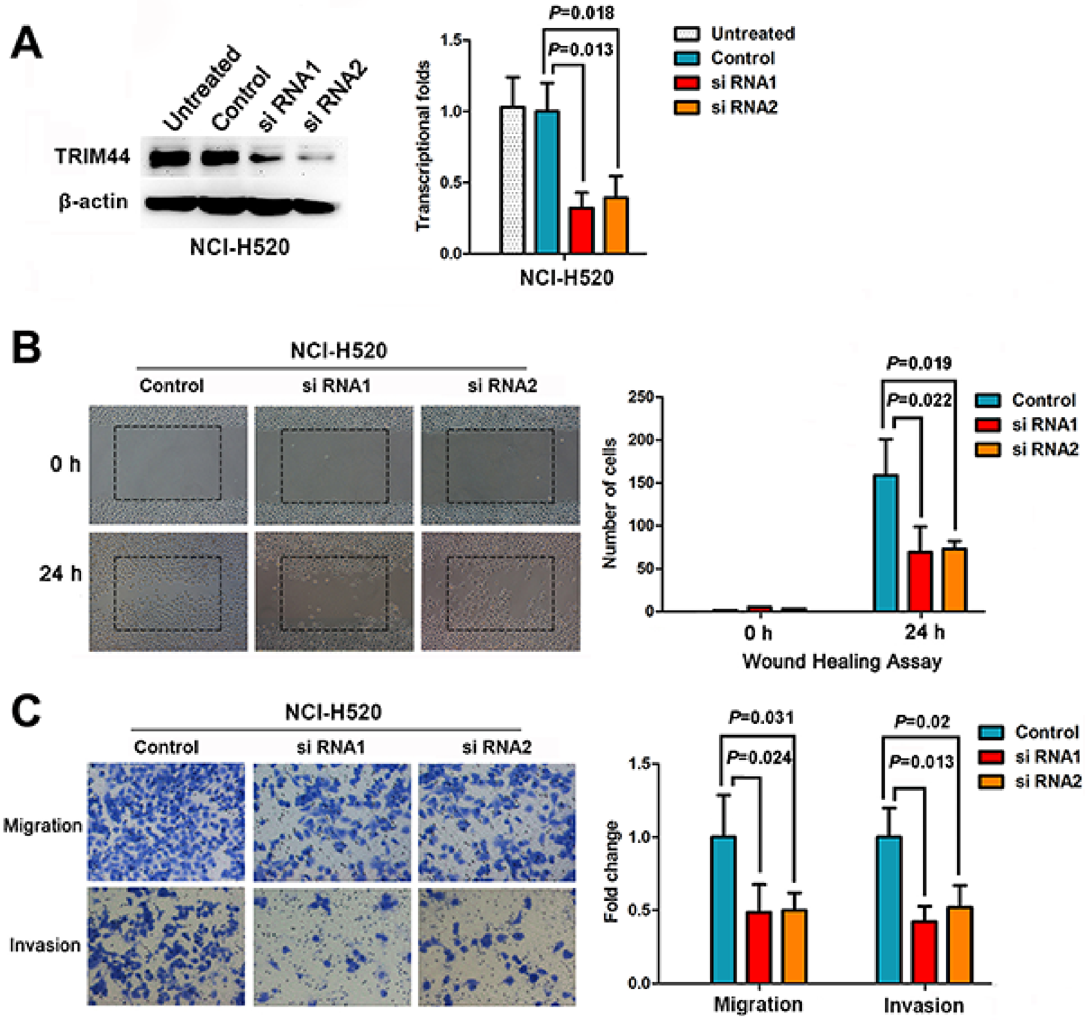

Supplementary Figure S3: Knockdown of TRIM44 in non-small lung cancer cells inhibits cell invasion and migration. (**A**) TRIM44 expression was confirmed by immunoblotting and real-time quantitative RT-PCR. TRIM44 expression in NCI-H520 cells was markedly reduced by RNA interference. (**B**) Wound healing assays to examine the migratory ability of NCI-H520 cells. (**C**) The invasion and migration of NCI-H520 cell lines and their derivatives was determined in a Transwell assay. Data are expressed as the mean ± SEM (n = 3). All P values were obtained using the Student's t-test. *$P < 0.05$.

Supplementary Table S1: Univariate and multivariate analyses of overall survival and disease-free survival

	OS			DFS		
	Univariate analysis	Multivariate analysis		Univariate analysis	Multivariate analysis	
Variable	*P*	HR (95% CI)	*P*	*P*	HR (95% CI)	*P*
Age						
< 60						
≥ 60	0.077	–	–	0.246	–	–
Gender						
Female						
Male	0.783	–	–	0.861	–	–
Differentiation						
Good		Ref				
Moderate		1.195 (0.726 to 1.967)	0.483			
Poor	0.018*	1.407 (0.850 to 2.327)	0.184	0.152	–	–
Histological cell type						
Squamous cell carcinoma		Ref			Ref	
Adenocarcinoma	0.036*	1.127 (0.812 to 1.564)	0.472	0.009*	1.185 (0.882 to1.590)	0.259
pTNM stage						
I		Ref			Ref	
II		1.500 (0.902 to2.495)	0.118		1.277 (0.825 to 1.976)	0.273
III/IV	< 0.001*	2.814 (1.460 to 5.423)	0.002*	< 0.001*	2.236 (1.260 to 3.968)	0.006*
Lymph node metastasis						
Present		Ref			Ref	
Absent	< 0.001*	1.288 (0.746 to 2.226)	0.363	< 0.001*	1.385 (0.858 to 2.237)	0.182
TRIM44 expression						
Low		Ref			Ref	
High	< 0.001*	1.695 (1.149 to 2.501)	0.008*	< 0.001*	1.737 (1.240 to 2.433)	0.001*

Abbreviations: NSCLC = non-small cell lung cancer; pTNM stage = Tumor, node, metastasis (pathological stage); pT = pathological T stage; *n* = number of patients; OS = overall survival; DFS = disease-free survival; HR = hazard ratio; CI = confidence interval. **P* < 0.05 was considered statistically significant.

Supplementary Table S2: Antibodies used for western blotting

Target	Dilution	Manufacturer
Pan mTOR	1:1000	Cell Signaling Technology
Phospho-mTOR (Ser2448)	1:1000	Cell Signaling Technology
Pan Akt	1:1000	Cell Signaling Technology
Phospho-Akt (Ser 473)	1:1000	Cell Signaling Technology
Phospho-P70S6K (Thr 389)	1:1000	Cell Signaling Technology
E-cadherin	1:2000	ProteinTech
N-cadherin	1:5000	Abcam
Vimentin	1:5000	Abcam
Cyclin D1	1:500	Santa Cruz Biotechnology
Cyclin E1	1:500	Cell Signaling Technology
CDK2	1:500	Santa Cruz Biotechnology
CDK4	1:500	Santa Cruz Biotechnology
TRIM44	1:1000	ProteinTech
β-actin	1:1000	Cell Signaling Technology
Mouse secondary antibody	1:10000	Zhong Shan Golden Bridge
Rabbit secondary antibody	1:10000	Zhong Shan Golden Bridge
